# Polyphenol-rich Morus nigra L. extract mitigates neuroinflammation and cognitive impairment through gut–brain axis modulation in an Alzheimer’s disease rat model

**DOI:** 10.3389/fphar.2025.1695768

**Published:** 2025-11-27

**Authors:** Yue Li, Yangyi Zhang, Mengwen Liu, Tuerxunayi Dawuti, Xuanshi Chen, Hui Xiao

**Affiliations:** 1 School of Public Health, Xinjiang Medical University, Urumqi, China; 2 Department of Clinical Nutrition, The First Affiliated Hospital of Xinjiang Medical University, Urumqi, China; 3 Health Management Center, The First Affiliated Hospital of Xinjiang Medical University, Urumqi, China; 4 Department of Clinical Nutrition, People’s Hospital of Xinjiang Uygur Autonomous Region, Urumqi, China; 5 The First People’s Hospital of Urumqi, Urumqi, China; 6 Department of Clinical Nutrition, The 904th Hospital of oint Logistics Support Force, Suzhou, China

**Keywords:** Morus nigra L., gut-brain axis, Alzheimer’s disease, gut microbiota, neuroinflammation, metabolomics

## Abstract

**Background:**

The gut–brain axis (GBA) has emerged as a critical pathway in the pathogenesis of Alzheimer’s disease (AD), offering a potential target for dietary interventions. This study aimed to explore the neuroprotective effects of a polyphenol-enriched extract from *Morus nigra L.* fruits (MMF) in an AD rat model, focusing on gut-brain communication.

**Methods:**

AD-like pathology was induced in rats using a combination of D-galactose and aluminum chloride, followed by a 10-week MMF treatment. Cognitive performance was evaluated using the Morris water maze, and brain Aβ_1_-_42_ accumulation and neuroinflammation (Iba1, GFAP) were assessed. Multi-omics approaches, including 16S rDNA sequencing and untargeted colonic metabolomics, were applied.

**Results:**

MMF treatment significantly enhanced spatial memory, reduced hippocampal Aβ burden, and attenuated glial activation. Furthermore, MMF restored gut microbial diversity and increased the abundance of short-chain fatty acid–producing Firmicutes taxa, which were inversely correlated with inflammation. Metabolomics analysis revealed that MMF modulated bile acid and lipid metabolic pathways, with β-muricholic acid, DHA, and ergosterol identified as key effectors.

**Conclusion:**

MMF alleviates AD pathology through modulation of the gut microbiota and metabolic reprogramming, suggesting a promising microbiota-targeted strategy for AD prevention.

## 1 Introduction

Alzheimer’s disease (AD) is a progressive and irreversible neurodegenerative disorder characterized by cognitive deterioration and functional decline. The disease typically progresses through three stages: preclinical AD, mild cognitive impairment (MCI), and clinical AD. The preclinical phase, which may span decades prior to symptom onset, is marked by pathological hallmarks including amyloid-β (Aβ) accumulation, tau hyperphosphorylation, and chronic neuroinflammation (Gustavsson et al., 2023). Although clinical symptoms are absent during this stage, these early pathological changes represent a critical window for intervention and are considered the optimal period for delaying disease progression (Rafii and Aisen, 2023; Cai et al., 2025). However, effective preventive strategies targeting this stage remain scarce, highlighting the urgent need for novel approaches.

Recent advances have identified the microbiota–gut–brain axis (GBA) as a key contributor to AD pathogenesis (Qu et al., 2024). Gut dysbiosis disrupts intestinal barrier function, facilitating systemic inflammation and the translocation of pro-inflammatory molecules into circulation. These circulating mediators may impair blood–brain barrier integrity, activate resident immune cells (particularly microglia), and aggravate neuroinflammatory cascades, Aβ aggregation, and tau pathology (Qu et al., 2024; Quigley, 2017; Chen et al., 2021; Dodiya et al., 2019; Wang et al., 2021; Zhuang et al., 2018; Wang et al., 2022a; Zhu et al., 2023). Both animal and clinical studies have demonstrated that alterations in gut microbial composition are associated with AD phenotypes, implicating the gut microbiota in modulating neuroinflammation and Aβ clearance (Wang et al., 2022a; Zhu et al., 2023). Consequently, the GBA has emerged as a promising target for therapeutic modulation to prevent or slow AD progression.

Natural products rich in bioactive phytochemicals have garnered attention as potential GBA modulators. Morus nigra L. (black mulberry), a traditional medicinal and dietary plant, has been used in ethnomedicine for its antioxidant and anti-inflammatory properties (Khalifa et al., 2018). Phytochemical analyses have identified a high content of polyphenolic compounds in Morus nigra, including anthocyanins, flavonoids, and resveratrol analogs, all of which exhibit potent biological activities. (Jiang and Nie, 2015; Jiang et al., 2011; Cásedas et al., 2024). Notably, dietary polyphenols have been shown to modulate gut microbiota composition and metabolic function, exerting prebiotic-like effects (Cheng et al., 2023; Xu and Zhang, 2022; Zhang et al., 2022). Given their limited systemic bioavailability, these compounds are believed to act primarily via the GBA, restoring microbial homeostasis and suppressing neuroinflammation to confer neuroprotection.

Further evidence supports the neuroprotective potential of Morus nigra-derived polyphenols. Experimental studies have demonstrated that mulberry extracts reduce levels of pro-inflammatory cytokines—such as IL-1β, TNF-α, nitric oxide (NO), and interferon-γ (IFN-γ)—in murine models (Chen H. et al., 2016). In D-galactose-induced cognitive impairment models, freeze-dried mulberry extract improved learning and memory, mitigated DNA damage, reduced malondialdehyde (MDA) levels, and enhanced antioxidant enzyme activities in the brain, liver, and serum (Turgut et al., 2016). Despite these promising results, the precise mechanisms by which Morus nigra exerts its neuroprotective effects remain incompletely understood. One major challenge in translating botanical polyphenols into brain-targeted therapies is their limited permeability across the blood–brain barrier. This limitation has spurred growing interest in leveraging the GBA as a more accessible route for delivering therapeutic effects.

In this study, we investigated the neuroprotective potential of a polyphenol-rich extract from Morus nigra L. fruits (MMF) as a preventive intervention in an AD rat model induced by D-galactose and aluminum chloride. We hypothesized that MMF alleviates cognitive deficits and neuropathological features by modulating the GBA. To explore this, we employed an integrative multi-omics approach combining 16S rDNA sequencing of gut microbiota with untargeted metabolomics of colonic contents. This systems-level strategy provides mechanistic insight into how MMF reshapes gut microbial ecology and host metabolism, offering a comprehensive understanding of its neuroprotective actions in AD.

## 2 Methods

### 2.1 Reagents and drug preparation

D-galactose (D-Gal) and aluminum chloride (AlCl_3_) were sourced from Sigma-Aldrich (St. Louis, MO, USA). Recombinant anti-Iba1 antibody (ab178846, 1:1000) was purchased from Abcam (Cambridge, UK), and the GFAP polyclonal antibody (16825-1-AP, 1:3000) was obtained from Proteintech Group, Inc. (Wuhan, China). The DAB chromogenic reagent kit (G1212) was supplied by Servicebio Technology Co., Ltd. (Wuhan, China). The enzyme-linked immunosorbent assay (ELISA) kit for rat Aβ_1_-_42_ detection was provided by Sangon Bioengineering Co., Ltd. (Shanghai, China). All other reagents were of analytical grade and were procured from commercial suppliers.

The dried fruits of Morus nigra L. (black mulberry) were obtained from Kuqa Pure Agriculture and Technology Development Co., Ltd. (Kuqa, Xinjiang, China). The botanical identity of the raw material was verified by Professor Haiyan Xu from the Chinese Medicinal Resources Laboratory, Xinjiang Medical University (Urumqi, China). A voucher specimen (Specimen No: XMU-MN-2023–007) has been deposited at −80 °C in the herbarium of the Collaborative Innovation Building (Room 1406), Xinjiang Medical University, for future reference. Upon receipt, the dried fruits were inspected for quality and confirmed to be free from foreign matter. The fruits were then ground into a fine powder using a commercial grinder and sieved through a 40-mesh sieve to ensure uniform particle size before extraction.

The polyphenol-rich extract was obtained from the mulberry powder via supercritical carbon dioxide (SC-CO2) extraction using an SFE120-50-05 supercritical fluid extraction system (Nantong Xinyanda Mechanical & Electrical Co., Jiangsu, China). Extraction was optimized with the following parameters: pressure of 35 MPa, temperature of 45 °C, and CO_2_ flow rate of 10–20 L/h, for a duration of 3–4 h. For every 300 g of mulberry powder, 100–200 mL of anhydrous ethanol was used as a co-solvent. After extraction, the ethanol was removed from the extract under reduced pressure, yielding a light-yellow crude extract. The extraction yield was 1.9% (w/w). The final extract, termed MMF, was stored in airtight, light-protected containers at −40 °C. The characterization of MMF adhered to the ConPhyMP guidelines (Heinrich and Jalil, 2023), and the completed checklist is included in the Supplementary Material.

### 2.2 Chemical composition analysis of MMF using UPLC-orbitrap-ms

The chemical composition of the mulberry extract was profiled using ultra-performance liquid chromatography coupled with a Q Exactive Orbitrap mass spectrometer (UPLC-Orbitrap-MS). The analysis was conducted in negative ion mode with a full mass scan range of m/z 100–900 at a resolution of 70,000. Chromatographic separation was achieved on a Waters HSS T3 column using a formic acid-acetonitrile/water gradient elution system. Flavonoid compounds were identified based on their accurate mass (mass error <10 ppm) and retention times, matched to authentic commercial standards. Quantification was carried out using external standard calibration curves to ensure the reliability and accuracy of metabolite concentrations.

### 2.3 Animals and experimental design

All experiments were conducted in accordance with the ARRIVE guidelines and were approved by the Animal Ethics Committee of Xinjiang Medical University (IACUC-20230110-10). Thirty male Sprague-Dawley rats (3 months old, 200–250 g) were obtained from the Experimental Animal Center of Xinjiang Medical University. The rats were housed under controlled conditions (22 °C ± 2 °C, 50% ± 10% humidity, 12-h light/dark cycle) with free access to standard food and water. The rats were randomly assigned to three groups (n = 10/group). During the study, two rats from each group were excluded due to severe health complications unrelated to the intervention, resulting in a final sample size of eight per group for all analyses. No replacements were made to maintain the integrity of random allocation and the treatment timeline.

An AD-like model was induced by D-galactose (D-Gal) and aluminum chloride (AlCl_3_), a well-established protocol that replicates key features of early AD pathogenesis, including cognitive decline and Aβ accumulation. The treatment groups were as follows (Gustavsson et al., 2023): Control: Saline +0.5% CMC-Na (Rafii and Aisen, 2023); Model: D-Gal (150 mg/kg, i. p.) + AlCl_3_ (20 mg/kg, p. o.) + 0.5% CMC-Na (Cai et al., 2025); MMF: D-Gal + AlCl_3_ + MMF (50 mg/kg, p. o.). Treatments were administered daily for 10 weeks, with the modeling agents given in the morning and MMF in the afternoon. The MMF dose (50 mg/kg/d, p. o.) was selected based on a previous dose-response study, which demonstrated superior efficacy in improving Morris water maze performance compared to 25 and 100 mg/kg/d.

After behavioral testing, the rats were anesthetized with 1% sodium pentobarbital (50 mg/kg, i. p.; equivalent to 0.5 mL/100 g body weight). Deep anesthesia was confirmed by the absence of corneal and paw withdrawal reflexes. Under deep anesthesia, the rats were euthanized by cervical dislocation, and samples were immediately collected. Brain tissues were snap-frozen for biochemical assays or fixed for histological analysis. Fecal samples were collected for microbiome analysis and stored at −80 °C.

To prevent bias, all procedures, including behavioral testing, histological evaluation, and data analysis, were performed by experimenters blinded to group assignments. Randomization for animal grouping was carried out using computer software to avoid any potential bias in treatment allocation.

### 2.4 Pharmacodynamic evaluation

Spatial learning and memory were assessed using the Morris Water Maze (MWM) test, comprising a spatial acquisition phase and a probe trial. During the acquisition phase (days 71–75), the rats underwent daily training for five consecutive days. On day 76, a probe trial was conducted to evaluate spatial memory retention.

Immunohistochemical (IHC) analysis was performed on coronal brain sections containing the hippocampus. Tissues were fixed in 4% paraformaldehyde for 48 h, dehydrated, embedded in paraffin, and sectioned at 5 μm thickness. Sections were treated with 0.5% hydrogen peroxide for 30 min to quench endogenous peroxidase activity, followed by blocking with 5% bovine serum albumin (BSA) for 1 h at room temperature to prevent nonspecific binding. Primary antibodies against Iba1 and GFAP were incubated overnight at 4 °C. After washing, secondary antibodies were applied for 1 h at 37 °C. Immunoreactivity was visualized using the DAB chromogenic reagent kit. For quantitative analysis, the hippocampal dentate gyrus (DG) subregion, known for its role in immune responses, was specifically examined. The stained sections were evaluated under a light microscope, and staining intensity was quantified using ImageJ software.

For ELISA analysis, brain tissues were rinsed with saline, weighed, and homogenized in ice-cold 0.86% saline at a tissue-to-solution ratio of 1:9 (w/v). The homogenates were centrifuged at 5,000 × g for 15 min at 4 °C, and the supernatants were collected. Aβ_1_–_42_ levels were measured using a commercial ELISA kit according to the manufacturer’s instructions.

### 2.5 16S rDNA gene sequencing of colon contents

Gut microbiota analysis of colon contents was performed by Novogene Co., Ltd. (Beijing, China) using the Illumina NovaSeq platform. DNA was extracted from fecal samples, and the 16S rDNA gene was amplified. Quality control was conducted using FLASH and fastp software. Chimera sequences were), and effective tags were retained. Taxonomic classification of 16S rDNA sequences was performed with Uparse software (v7.0.1001, http://drive5.com/uparse/). Functional gene enrichment was analyzed using the KEGG database, and data visualization was performed on the NovoMagic platform (https://magic.novogene.com/). Correlations between bacterial relative abundances and other experimental results were analyzed using Spearman correlation and displayed as a heatmap in R (V4.2.1).

### 2.6 Untargeted metabolomics analysis of colon contents

Metabolomic profiling was performed using a Vanquish UHPLC system (Thermo Fisher, Germany) coupled with an Orbitrap Q Exactive™ HF or HF-X mass spectrometer (Thermo Fisher, Germany) at Novogene Co., Ltd. Raw data from UHPLC-MS/MS were processed with Metabolite Discoverer 3.3 (CD3.3, Thermo Fisher) for peak alignment, picking, and quantification. To ensure data quality, metabolites with a coefficient of variation (CV) > 30% in quality control (QC) samples were excluded from further analysis. Differential metabolites were identified based on a VIP >1 and *p* < 0.05, with annotations performed using the KEGG, HMDB, and LIPID MAPS databases. Traceability analysis of differential metabolites was conducted using MetOrigin (http://metorigin.met-bioinformatics.cn/). Functional and origin analyses, as well as Sankey network visualizations, were performed using the simple MetOrigin analysis mode.

### 2.7 Statistical analysis

All quantitative data are presented as mean ± standard error of the mean (SEM). Statistical analyses were performed using GraphPad Prism 9.5.1. Normality of data was assessed using the Shapiro-Wilk test, and variance homogeneity was evaluated with Levene’s test. One-way ANOVA, followed by Tukey’s *post hoc* test, was used to compare group differences. For multiple comparisons, the false discovery rate (FDR) correction was applied to control for type I errors. Statistical significance was defined as *p* < 0.05, and effect sizes and confidence intervals were reported where applicable.

## 3 Results

### 3.1 Chemical identification of MMF

UPLC-QE-MS analysis confirmed that MMF is a complex mixture comprising 30 identified phenolic compounds (Supplementary Figure S1). The chemical composition was quantified using external standard calibration curves, and the concentrations of identified compounds are presented in Supplementary Table S1. This profile includes several bioactive phytochemicals with known neuroprotective properties, such as epicatechin, resveratrol, quercetin, luteolin, and apigenin (Chiang et al., 2023; Ozpak and Bağca, 2024; Vongthip et al., 2024; Wu et al., 2024; Yoon et al., 2025). The simultaneous presence of these phenolics and flavonoids highlights the extract’s potential for multi-target action and provides a solid chemical foundation for subsequent pharmacological investigations.

### 3.2 MMF alleviated cognitive impairment and histopathological changes in AD rats

**FIGURE 1:**
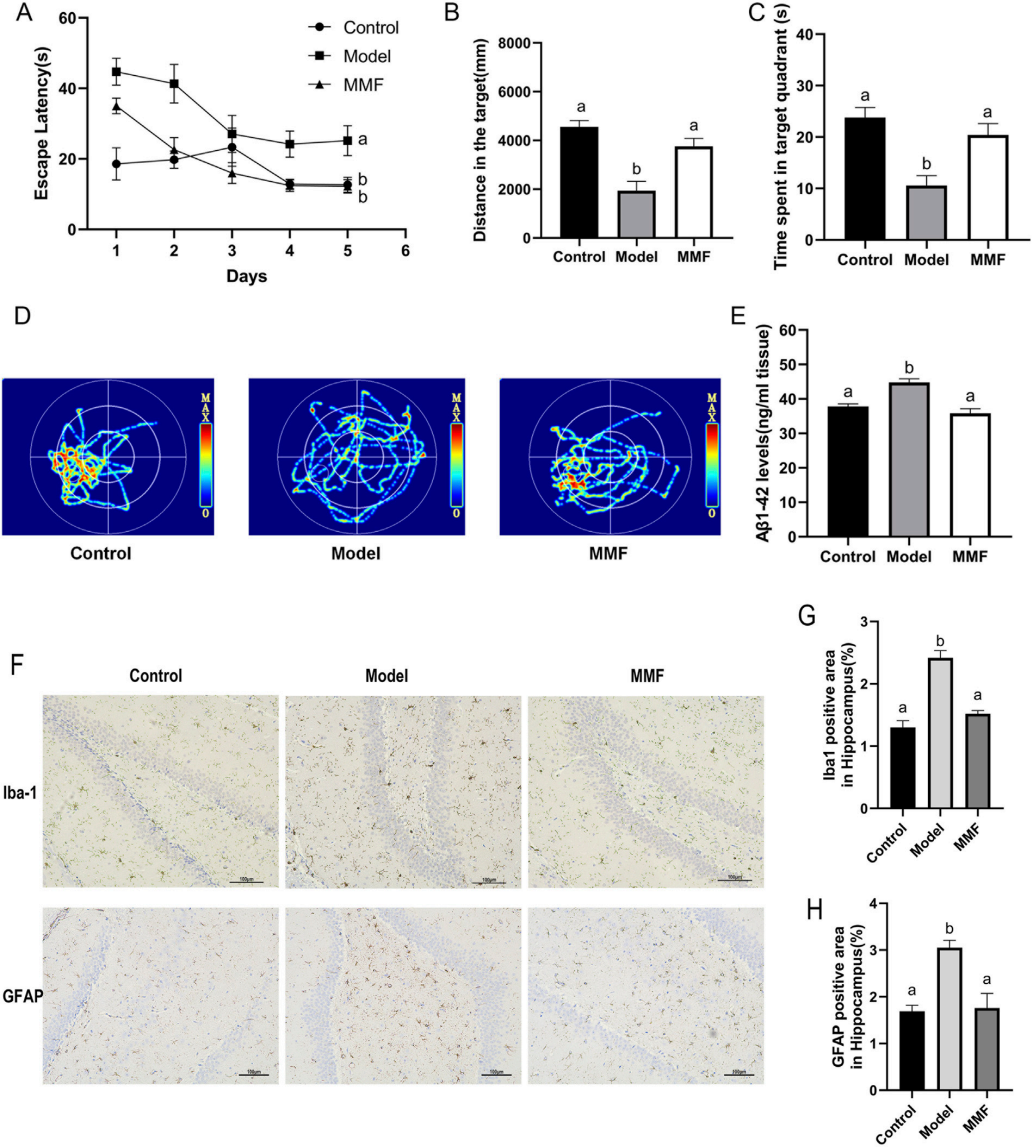
Pharmacodynamic evaluation of MMF against AD. **(A)** Escape latency in the training trial test. **(B)** Travel distance of rats in the target quadrant during the probe trial. **(C)** Time spent in the target quadrant. **(D)** Representative images of swim paths for different groups in the probe test. **(E)** Levels of Aβ_1_–_42_ in the brain. **(F)** Representative IHC images of Iba1 and GFAP in the hippocampal dentate gyrus (DG). **(G)** Proportion of Iba1 positive area in hippocampus. **(H)** Proportion of GFAP positive area in hippocampus. Data are presented as mean ± SEM. Compared to the model group, different superscripts **(A, B)** indicate significant statistical differences (*p* < 0.05), n = 6.

In the Morris water maze test, rats in the AD model group exhibited significant cognitive impairments, as evidenced by prolonged escape latency and reduced exploration of the target quadrant (Figures 1A–C). Furthermore, Aβ_1_–_42_ levels were significantly elevated (Figure 1E), confirming the successful establishment of the AD-like model. MMF treatment significantly reduced escape latency and increased swimming distance and time spent in the target quadrant during the probe trial (Figures 1A–C). Additionally, MMF-treated rats exhibited more platform crossings (Figure 1D), suggesting an improvement in spatial memory.

MMF treatment effectively suppressed the activation of Iba-1^+^ microglia and GFAP^+^ astrocytes in the hippocampus (Figures 1F–H), key cellular mediators of neuroinflammation in AD, consistent with a reduction in neuroinflammation. Furthermore, MMF significantly decreased Aβ_1_–_42_ levels (Figure 1E), demonstrating its potential to mitigate amyloid pathology.

### 3.3 16S rDNA sequencing analysis of gut microbiota

#### 3.3.1 OTU analysis

Operational Taxonomic Unit (OTU) analysis at various taxonomic levels (phylum to species; Supplementary Table S2) revealed a significant reduction in OTU counts in the model group compared to controls, indicating dysbiosis of the gut microbiota. MMF treatment partially restored OTU abundance (Supplementary Figure S2A), suggesting a potential restorative effect on microbial composition.

#### 3.3.2 Alpha diversity

**FIGURE 2:**
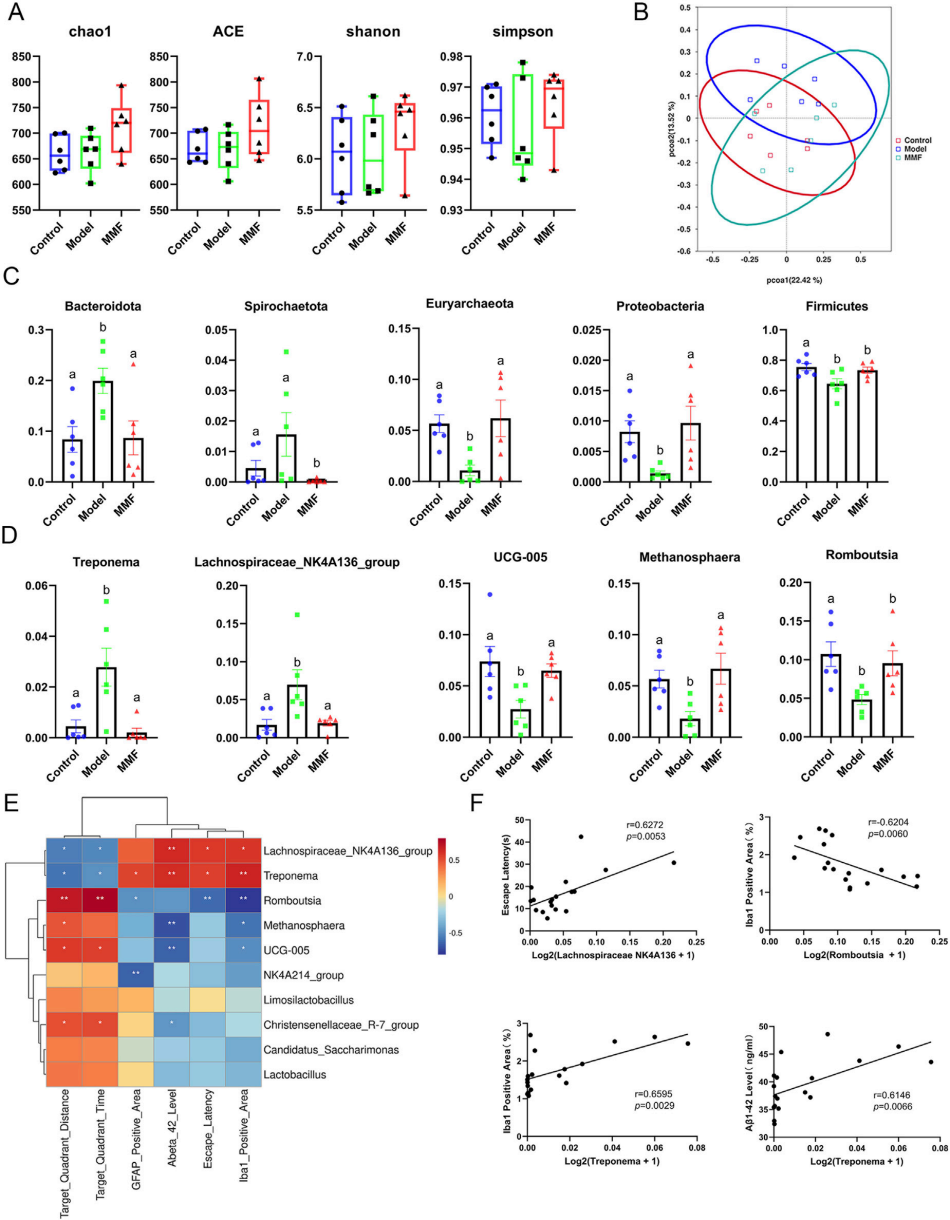
Diversity and differential analyses of gut microbiota. **(A)** Alpha diversity analysis. **(B)** PCoA score plot of all experimental groups. **(C)** Differential microbiota at the phylum level. **(D)** Differential microbiota at the genus level. **(E)** Heatmap of the association between microbiota and other experimental results. **(F)** Scatter plot of the association between microbiota and other experimental results. (Control: Control group; Model: Model group; MMF: MMF-treated group. Data are presented as mean ± S.E. Compared to the model group, different superscripts **(A, B)** indicate significant statistical differences (*p* < 0.05), n = 6.

Alpha diversity was assessed using Chao1, Ace, Shannon, and Simpson indices (Figure 2A). No significant differences were observed between the MMF and model groups; however, MMF treatment showed a trend toward the recovery of microbial richness and diversity.

#### 3.3.3 Beta diversity

Principal Coordinates Analysis (PCoA) of beta diversity demonstrated a clear separation between the control and model groups. The MMF group exhibited a shift in microbiota composition toward that of the control group (Figure 2B), indicating partial restoration of the microbiota structure in AD rats.

#### 3.3.4 Microbial composition and differential abundance

At the phylum level (Figure 2C), the model group exhibited increased levels of Bacteroidota and Spirochaetota, and decreased levels of Firmicutes, Euryarchaeota, and Proteobacteria. MMF treatment reversed these alterations.

At the genus level (Figure 2D), *Treponema* and Lachnospiraceae_NK4A136_group were elevated in the model group, while Methanosphaera, Romboutsia, and UCG-005 were reduced. MMF treatment restored these genera to control levels, suggesting normalization of microbial dysbiosis.

#### 3.3.5 Microbiota–pathology correlations

Significant correlations were observed between bacterial genera and pathological indices (Figures 2E,F). The abundance of Romboutsia was negatively correlated with the Iba-1-positive microglial area (r = −0.6204, p = 0.0060), while *Treponema* abundance was positively correlated with both the Iba-1-positive area (r = 0.6595, p = 0.0029) and Aβ_1_–_42_ levels (r = 0.6146, p = 0.0066). Lachnospiraceae_NK4A136_group positively correlated with escape latency (r = 0.6272, p = 0.0053). These statistically significant correlations suggest that specific gut bacterial genera may mediate the relationship between gut dysbiosis and brain pathology, providing a mechanistic basis for the protective effects of MMF.

#### 3.3.6 Functional profiling of microbiota

Principal Component Analysis (PCA) of KEGG-annotated functional pathways showed clear separation between the control and model groups (Supplementary Figure S2B). MMF treatment shifted the functional profile toward that of the control state. Among 48 altered metabolic pathways in the model group, MMF modulated 25 pathways, including key pathways such as glycolysis, chorismate metabolism, TCA cycle, and the glyoxylate bypass (Supplementary Figures S2C,D).

### 3.4 Metabolomics analysis of MMF against AD

#### 3.4.1 PLS-DA of experimental groups

**FIGURE 3:**
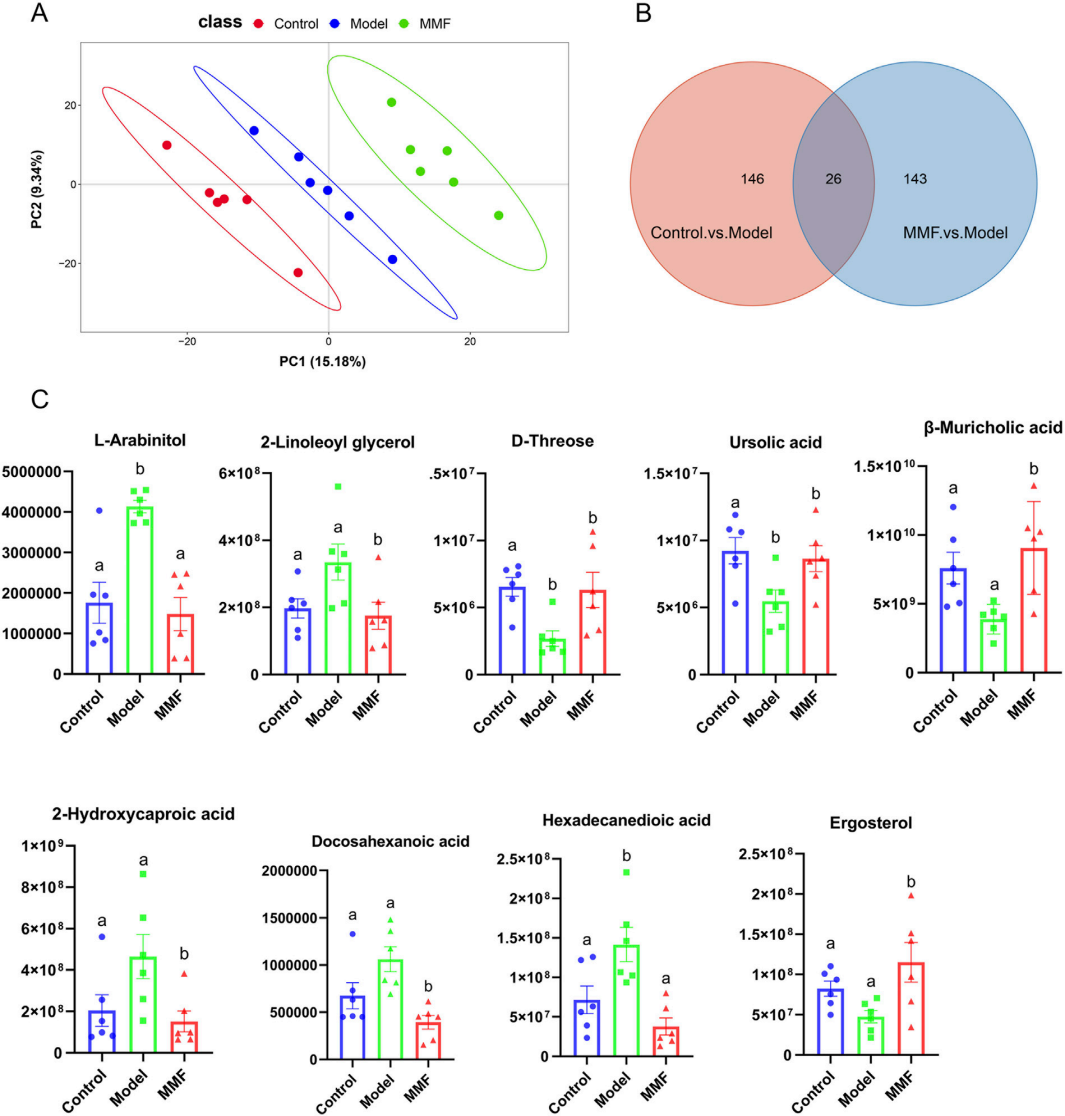
Metabolomics multivariate statistical analysis and differential metabolites. **(A)** PLS-DA score plot for all experimental groups. **(B)** Venn diagram of common differential metabolites among the three groups. **(C)** Diagram of relative abundance of different metabolites in colonic contents. (Control: Control group; Model: Model group; MMF: MMF-treated group. Data are presented as mean ± SEM. Compared to the model group, different superscripts **(A, B)** indicate significant statistical differences (*p* < 0.05, n = 6.).

Quality control (QC) samples demonstrated high reproducibility (Pearson correlation coefficients ≈1; Supplementary Figure S3A), confirming the robustness of the analytical platform. Partial least squares-discriminant analysis (PLS-DA) of fecal metabolomes revealed distinct separations among the control, model, and MMF-treated groups (Figure 3A), reflecting characteristic metabolic profiles and the modulatory role of MMF. The PLS-DA model exhibited strong predictive reliability (Q^2^Y > 0.5) without evidence of overfitting, as validated by permutation testing (Q^2^ intercept <0; Supplementary Figure S3B). Differential metabolites were selected based on variable importance in projection (VIP) > 1 and *p* < 0.05.

#### 3.4.2 Regulation of differential metabolites by MMF

Compared with controls, the AD model group exhibited 172 significantly altered metabolites (128 upregulated and 44 downregulated). MMF treatment substantially modulated 169 metabolites relative to the model group (75 upregulated, 94 downregulated; Supplementary Figure S3C,D). Twenty-six metabolites overlapped between these comparisons (Figure 3B), of which nine were strongly associated with AD pathology based on ROC curve analysis and MetAnnotation results (Table 1; Figure 3C).

**TABLE 1:** Common differential metabolites in colonic contents of three groups of rats.

NO.	Name	HMDB_ID	Formula	RT [min]	m/z	ROC	VIP
1	D-threose	HMDB0250746	C4 H8 O4	5.989	285.081	0.972	2.125
2	β-Muricholic acid	HMDB0000415	C24 H40 O5	6.667	407.280	0.944	1.293
3	L-arabinitol	HMDB0001851	C5 H12 O5	1.291	175.058	0.917	1.545
4	Ursolic acid	HMDB0002395	C30 H48 O3	10.389	455.354	0.861	1.687
5	Ergosterol	HMDB0000878	C28 H44 O	10.351	397.347	0.889	1.129
6	Hexadecanedioic acid	HMDB0000672	C16 H30 O4	7.579	285.208	0.833	1.494
7	2-Linoleoyl glycerol	HMDB0245187	C21 H38 O4	10.116	337.271	0.833	1.061
8	2-Hydroxycaproic acid	HMDB0001624	C6 H12 O3	5.618	131.072	0.861	1.498
9	Docosahexanoic acid	HMDB0002183	C22 H32 O2	10.428	327.233	0.861	1.037

In AD model rats, metabolic perturbations were characterized by elevated levels of L-arabinitol and hexadecanedioic acid, and decreased levels of D-threose and ursolic acid. MMF treatment significantly normalized seven key metabolites related to glucose metabolism, lipid regulation, and inflammation—β-muricholic acid, L-arabinitol, hexadecanedioic acid, 2-linoleoyl glycerol, 2-hydroxycaproic acid, docosahexaenoic acid (DHA), and ergosterol—indicating a restorative effect on metabolic homeostasis.

#### 3.4.3 MetOrigin tracing and metabolic pathway analysis

**FIGURE 4:**
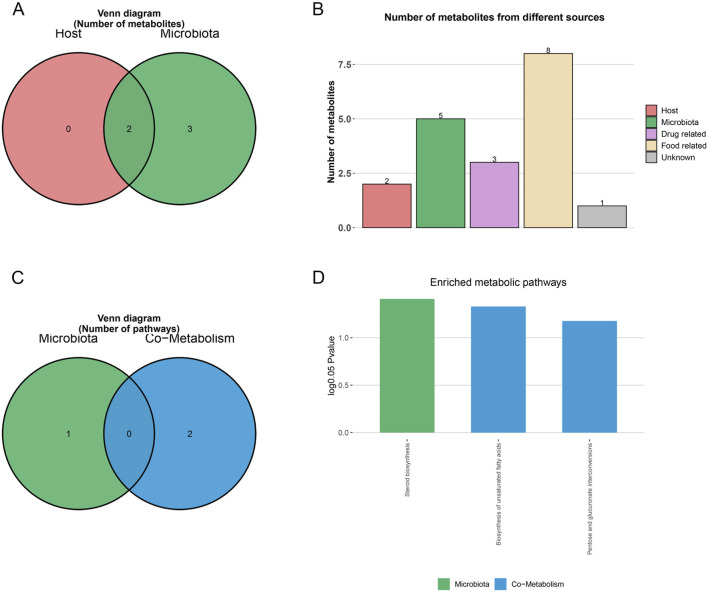
MetOrigin metabolite traceability analysis of differential metabolites. **(A,B)** Venn diagram and histogram of differential metabolites. **(C,D)** Venn diagram and histogram of enrichment analysis of differential metabolic pathways.

MetOrigin analysis classified the nine MMF-regulated, AD-associated metabolites according to their biological origin: two were bacterial–host co-metabolites, three were exclusively microbial, and four were derived from other sources, such as diet or xenobiotics (Figures 4A,B). Subsequent metabolic pathway enrichment analysis (MPEA) linked these metabolites to pathways of distinct origins: steroid biosynthesis (microbial origin), biosynthesis of unsaturated fatty acids, and pentose/glucuronate interconversions (co-metabolic pathways) (Figure 4C). MMF significantly modulated all three AD-related pathways (Figure 4D), suggesting its dual capacity to influence both microbial and host metabolic processes. Notably, MetOrigin analysis revealed that the MMF-modulated metabolic pathways were predominantly associated with fungal taxa (Ascomycota and Basidiomycota), suggesting a previously unrecognized fungal-associated remodeling of gut metabolic ecology as a novel mechanism through which MMF may exert its anti-AD effects.

#### 3.4.4 Microbial–metabolite correlation networks

**FIGURE 5:**
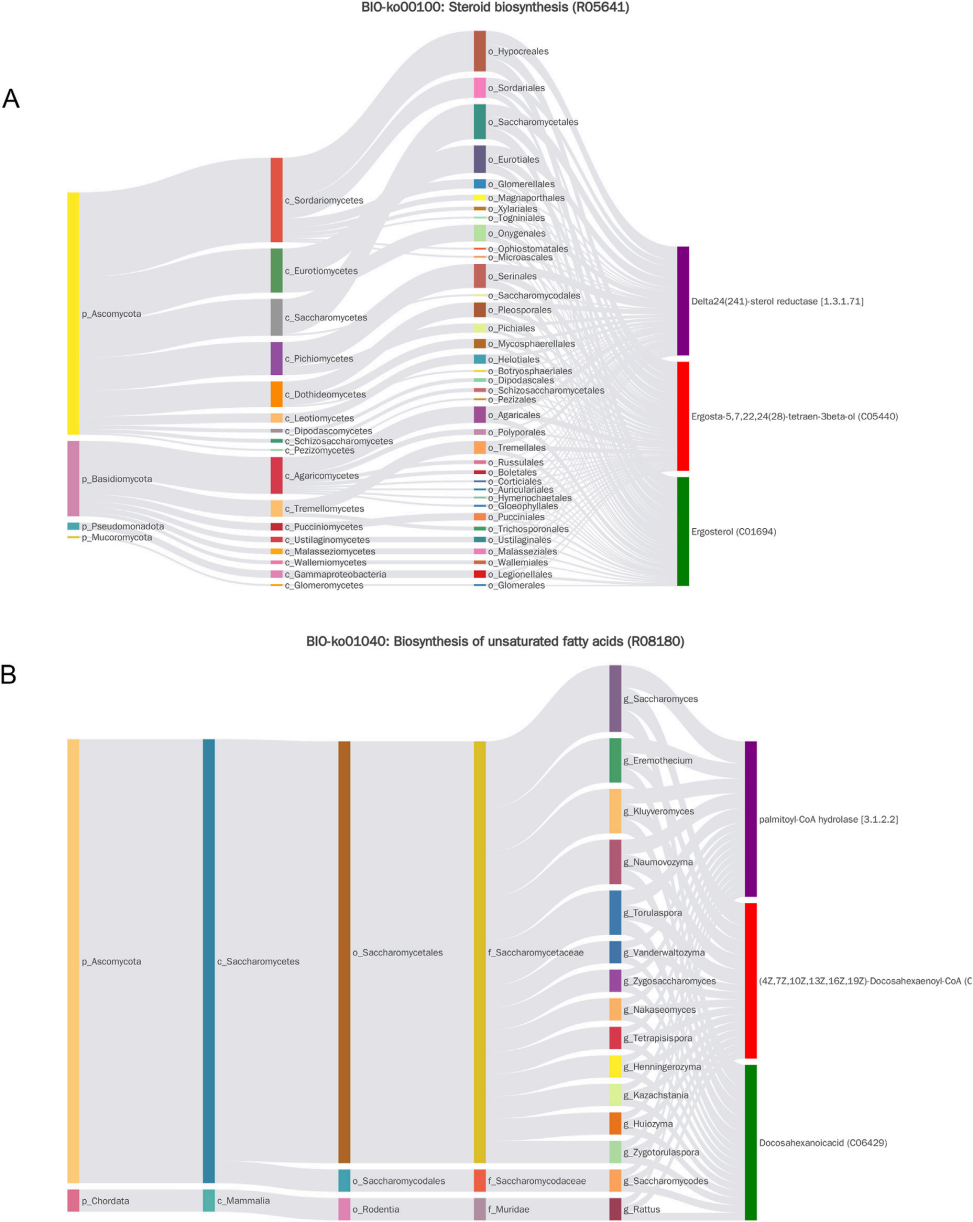
Sankey diagram of MetOrigin analysis. **(A)** Sankey diagram of steroid biosynthesis. **(B)** Sankey diagram depicting the biosynthesis of unsaturated fatty acids.

Bio-Sankey network visualization revealed strong correlations between microbial taxa and metabolites identified through MetOrigin. Key associations included: steroid biosynthesis—conversion of ergosta-5,7,22,24 (28)-tetraen-3β-ol to ergosterol (reaction R05641), primarily associated with Ascomycota and Basidiomycota; unsaturated fatty acid biosynthesis—production of docosahexaenoic acid (DHA) (reaction R08180), predominantly linked to Ascomycota; and pentose and glucuronate interconversions—involvement of L-arabinitol in multiple reactions (R01758, R01759, R01903), facilitated by D-xylose reductase and strongly correlated with Ascomycota and Basidiomycota (Figures 5A,B; Supplementary Figures S4A-C). These findings suggest an association between MMF’s effects and fungal metabolic activity within the gut, indicating a potential mechanism through which MMF ameliorates AD pathology by enhancing the production of beneficial metabolites.

## 4 Discussion

This study demonstrates that early intervention with a polyphenol-rich mulberry fruit extract (MMF) alleviates Alzheimer’s disease (AD) pathology in a rat model through modulation of the gut-brain axis (GBA). Our findings demonstrate that early MMF intervention significantly improved cognitive performance and spatial memory, while concurrently alleviating key pathological brain changes, including reduced Aβ_1_–_42_ accumulation and downregulation of neuroinflammatory markers Iba-1 and GFAP. These robust pharmacological outcomes underscore MMF’s potential as a multitarget preventive agent against AD.

Key mechanistic insights into MMF’s action were derived from integrated analyses of gut microbiota and metabolomics data, which collectively suggest that MMF mitigates AD pathology through modulation of the GBA axis. Microbiota profiling revealed significant dysbiosis in AD rats, characterized by an increased abundance of Bacteroidota and a decrease in Firmicutes, a pattern consistent with clinical observations in AD patients (Vogt et al., 2017). This imbalance, exacerbated by aging and linked to immune dysfunction and chronic inflammation (Vogt et al., 2017; Odamaki et al., 2016; Wang SS. et al., 2022), was effectively restored by MMF treatment. Specifically, MMF increased the abundance of Firmicutes, particularly beneficial genera such as Romboutsia and Ruminococcaceae_UCG-005, known for their anti-inflammatory properties (Rosell-Díaz et al., 2024; Shi et al., 2023).

The functional significance of this microbial restoration is noteworthy. Romboutsia and Ruminococcaceae_UCG-005 are essential for fermenting dietary fibers into short-chain fatty acids (SCFAs) such as butyrate, which exerts potent anti-inflammatory effects, strengthens intestinal and blood-brain barriers, and reduces the systemic translocation of pro-inflammatory molecules like LPS (Kelly et al., 2015; Vi et al., 2017; Wang et al., 2022c). This suppression of peripheral inflammation attenuates excessive microglial activation and neuroinflammation. Furthermore, MMF regulates microbial metabolic pathways, including glycolysis and the tricarboxylic acid (TCA) cycle, which are implicated in Aβ aggregation, tau pathology, and mitochondrial dysfunction in AD (Zhang et al., 2021; Andreyev et al., 2024). Together, our *in vivo* efficacy data and multi-omics profiling suggest that MMF may alleviate AD symptoms by modulating the gut-brain axis through restoration of microbial homeostasis and enhancement of SCFA-related anti-inflammatory and metabolic functions.

Building on the observed microbiota remodeling, our metabolomic analysis further elucidates the microbial-mediated mechanisms underlying MMF’s therapeutic effects. MMF not only links gut microbiota modulation to the amelioration of AD but also provides direct metabolomic evidence of its microbial-driven actions. In addition to attenuating neuroinflammation, MMF corrected disturbances in glucose and lipid metabolism—key hallmarks of AD (Di Paolo and Kim, 2011; Tan et al., 2020; Cunnane et al., 2020). Several metabolites, including D-threose, ursolic acid, β-muricholic acid, L-arabinitol, and DHA, were significantly altered in AD rats and restored by MMF. These compounds are involved in steroid and unsaturated fatty acid biosynthesis, as well as pentose and glucuronate interconversions. Notably, the normalization of L-arabinitol highlights MMF’s ability to restore core metabolic pathways (Chen J. et al., 2016; Lin et al., 2013; Shang et al., 2015; Yu et al., 2017). These metabolic changes, tightly linked to the gut microbiota, underscore the pivotal role of the gut-brain axis (GBA). MMF specifically restored β-muricholic acid—a secondary bile acid synthesized through microbial metabolism (Pan et al., 2017)—suggesting a direct pathway through which MMF may enhance lipid metabolism and suppress neuroinflammation. Similarly, the modulation of ursolic acid, ergosterol, and DHA suggests that MMF may alleviate oxidative stress through a microbiota–metabolite network.

Most strikingly, MetOrigin analysis traced the origin of key neuroprotective metabolites, such as ergosterol and DHA, not just to the bacterial community, but specifically to fungal taxa, primarily Ascomycota and Basidiomycota (Figure 5). This suggests that MMF reshapes microbial metabolic output beyond taxonomic associations. These microbial-derived metabolites act as mediators of gut-brain communication; for example, β-muricholic acid influences systemic immunity and neuroinflammation via FXR and TGR5 signaling (Hong et al., 2023), while DHA and ursolic acid offer neuroprotection and reduce oxidative stress (Kim et al., 2022; Mirza and Zahid, 2022). In summary, MMF remodels the gut microbiota by enhancing beneficial Firmicutes and fungi, thereby altering metabolic output (e.g., DHA, ergosterol, β-muricholic acid, and ursolic acid). These metabolites, in turn, mitigate neuroinflammation, oxidative stress, and metabolic dysfunction, culminating in improved cognitive function in AD. These results suggest a potential paradigm shift from a purely bacterial perspective of the gut-brain axis to one that incorporates fungi as key metabolic players in mediating the benefits of polyphenol-rich interventions.

Our findings position MMF as a multifaceted preventive agent targeting key pathological features of AD through regulation of the GBA. The growing body of evidence supporting dietary interventions targeting gut microbiota for AD prevention further strengthens our results. For example, a recent study demonstrated that hydrogen-rich water alleviated cognitive decline and AD pathology in a zebrafish model, with restoration of beneficial gut microbiota (He et al., 2024). This convergence of evidence underscores the potential of targeting gut dysbiosis as a preventive strategy for AD. MMF’s restoration of microbial ecology, characterized by the enrichment of beneficial genera, enhances their functional metabolic capacity. This was demonstrated by the significant modulation of key microbial-centric pathways, such as steroid biosynthesis and the biosynthesis of unsaturated fatty acids. The subsequent increase in beneficial microbial metabolites and the restoration of metabolic homeostasis are posited to be the key mediators that attenuate both systemic and neuroinflammation. Therefore, we propose a prebiotic-like mechanism for MMF, wherein it directly modulates gut-derived neuroprotective metabolites that exert systemic effects on the host, ultimately contributing to the alleviation of AD pathology.

This study also generates important hypotheses regarding the multitarget and multipathway mechanisms by which MMF alleviates AD pathology. Several limitations must be acknowledged. The D-galactose/AlCl_3_-induced rat model, while effective at recapitulating key AD features like cognitive impairment and Aβ deposition, does not fully replicate the complexity of human AD (Zhang et al., 2020). Moreover, while multi-omics analyses revealed potential links between MMF and numerous targets and pathways, causal relationships remain to be established. In particular, the role of gut microbiota-derived metabolites in modulating AD via the gut-brain axis warrants further investigation (Doifode et al., 2021). It is important to acknowledge a primary limitation of this study: while our integrated multi-omics approach reveals compelling associations between MMF intervention, gut ecosystem remodeling, and amelioration of AD pathology, it does not establish direct causality. To move beyond these correlative findings, the critical next step is to demonstrate functional engagement of the gut-brain axis *in vivo*. Future studies employing fecal microbiota transplantation (FMT) from MMF-treated donors to germ-free or antibiotic-treated AD model recipients would be pivotal. A significant attenuation of MMF’s cognitive benefits and neuropathological alleviation in the recipient animals would provide definitive evidence that the reshaped gut microbiome is a necessary mediator of its therapeutic effects. Furthermore, to dissect the specific role of the fungal-driven metabolic pathways identified here, investigations using transgenic models or targeted inhibition of key metabolites and their receptors are essential to establish the causal mechanistic links.

In conclusion, our integrated multi-omics approach indicates that the preventive efficacy of MMF against AD is intricately linked to its prebiotic-like capacity to remodel the gut ecosystem, with a notable role for fungal-driven metabolic reprogramming. These findings not only position MMF as a promising candidate for functional food development but, more importantly, highlight the gut microbiome, particularly the mycobiome, as a fertile ground for discovering novel mechanisms and therapeutic strategies against neurodegenerative diseases.

## 5 Conclusion

This study identifies MMF as a promising multi-target dietary intervention for the early prevention of AD. Using a D-galactose and aluminum chloride-induced rat model, we demonstrate that MMF significantly ameliorates cognitive impairments and core AD pathologies, including Aβ deposition and neuroinflammation. Through integrated multi-omics analyses, we show that MMF exerts its therapeutic effects primarily by modulating the gut–brain axis. Specifically, MMF restores gut microbial homeostasis by enriching beneficial bacterial taxa and enhancing the metabolic activity of fungal communities, notably Ascomycota and Basidiomycota. This microbiota remodeling induces a shift in host metabolism, modulating the biosynthesis of neuroprotective metabolites such as docosahexaenoic acid, ergosterol, and β-muricholic acid. Together, these findings position MMF as a potential natural therapeutic agent for AD and underscore the gut mycobiome as an emerging target in neurodegenerative disease intervention. Furthermore, MMF supports the development of functional food strategies aimed at preventing AD, offering a safe, sustainable, and accessible approach to early intervention.

## Data Availability

The raw data supporting the conclusions of this article will be made available by the authors, without undue reservation.
