# Supplementary material for: Polyphenol-rich Morus nigra L. extract mitigates neuroinflammation and cognitive impairment through gut–brain axis modulation in an Alzheimer’s disease rat model
Source: Front Pharmacol. 2025 Nov 27;16:1695768. doi: 10.3389/fphar.2025.1695768 (PMC12695808; doi:10.3389/fphar.2025.1695768)
Supplement: Supplementary file 2 [file DataSheet3.docx]

Supporting Information

**Supplementary files**

**Figure S1.** LC-MS chromatogram of MMF. The peak numbers correspond to those listed in Table S1.

**Figure S2.** Diversity and functional analyses of gut microbiota. (A) Statistical histogram of OTU. (B) Microbial functional distance matrix combined with PCA. (C) Differences in microbial metabolic pathways between Model group and Control group. (D) Differences in microbial metabolic pathways between the MMF group and Model group.

**Figure S3.** Metabolomics supplement results. (A) Pearson correlation coefficients among QC samples. (B) Model validation diagram of PLS-DA score plot for all experimental groups. (C) Volcano plot of differential metabolites between the Control and Model groups. (D) Volcano plot of differential metabolites between the MMF and Model groups.

**Figure S4.** Sankey Diagram of Pentose and Glucuronate Interconversions. (A) Reaction R01758. (B) Reaction R01759. (C) Reaction R01903.

**Table S1.** Chemical composition of MMF based on UPLC-QE-MS.

**Table S2.** Statistical analysis of the number of microbial OTUs at various levels.


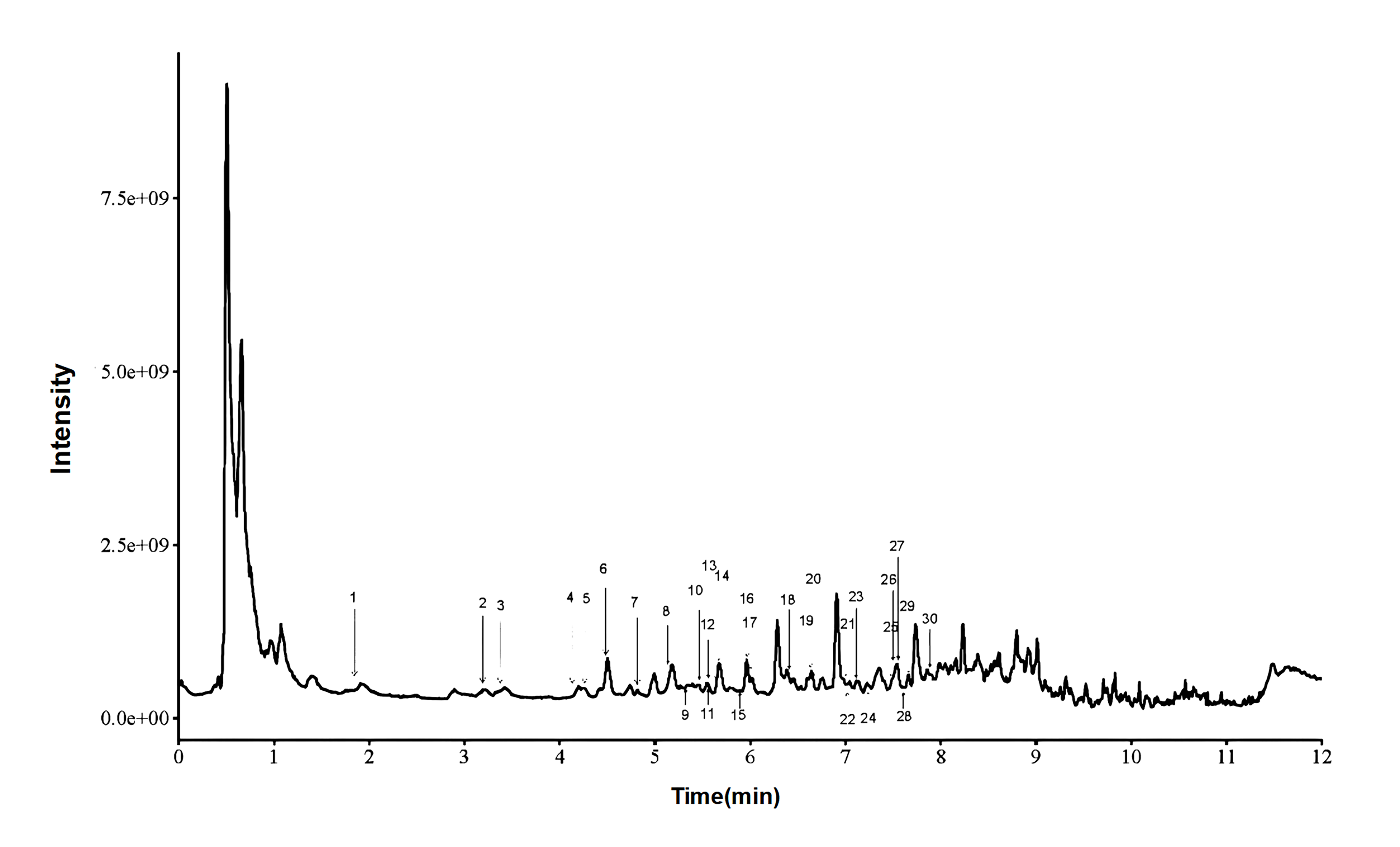


Figure S1. LC-MS chromatogram of MMF. The peak numbers correspond to those listed in Table S1.





**Figure S2.** Diversity and functional analyses of gut microbiota. (A) Statistical histogram of OTU. (B) Microbial functional distance matrix combined with PCA. (C) Differences in microbial metabolic pathways between Model group and Control group. (D) Differences in microbial metabolic pathways between the MMF group and Model group.





**Figure S3.** Metabolomics supplement results. (A) Pearson correlation coefficients among QC samples. (B) Model validation diagram of PLS-DA score plot for all experimental groups. (C) Volcano plot of differential metabolites between the Control and Model groups. (D) Volcano plot of differential metabolites between the MMF and Model groups.








**Figure S4.** Sankey Diagram of Pentose and Glucuronate Interconversions. (A) Reaction R01758. (B) Reaction R01759. (C) Reaction R01903.

**Table S1. Chemical composition of MMF based on UPLC-QE-MS**

| ID. | Compound | Formula | CAS | Rt/min | Mass fraction (μg/g) |
| --- | --- | --- | --- | --- | --- |
| 1 | 3,4-Dihydroxybenzoic acid | C7H6O4 | 99-50-3 | 1.93 | 10.21087206 |
| 2 | Protocatechualdehyde | C7H6O3 | 139-85-5 | 3.23 | 3.913968546 |
| 3 | 4-Hydroxybenzoic acid | C7H6O3 | 99-96-7 | 3.44 | 8.757472861 |
| 4 | Vanillic acid | C8H8O4 | 121-34-6 | 4.2 | 8.757899299 |
| 5 | Caffeic acid | C9H8O4 | 331-39-5 | 4.36 | 0.189629205 |
| 6 | Syringic acid | C9H10O5 | 530-57-4 | 4.55 | 0.983832099 |
| 7 | Epicatechin | C15H14O6 | 490-46-0 | 4.82 | 0.004457659 |
| 8 | Vanillin | C8H8O3 | 121-33-5 | 5.17 | 1.355993365 |
| 9 | p-Hydroxycinnamic Acid | C9H8O3 | 501-98-4 | 5.33 | 1.760112931 |
| 10 | Syringaldehyde | C9H10O4 | 134-96-3 | 5.49 | 0.321786018 |
| 11 | Salicylic acid | C7H6O3 | 69-72-7 | 5.6 | 0.548460533 |
| 12 | Vitexin | C21H20O10 | 3681-93-4 | 5.62 | 0.004074215 |
| 13 | Trans-Ferulic acid | C10H10O4 | 537-98-4 | 5.67 | 1.841068605 |
| 14 | Sinapic Acid | C11H12O5 | 530-59-6 | 5.71 | 2.118392436 |
| 15 | (+)-Dihydroquercetin | C15H12O7 | 480-18-2 | 5.85 | 0.073927768 |
| 16 | Benzoic acid | C7H6O2 | 65-85-0 | 6.05 | 4.144826788 |
| 17 | Kaempferol-3-O-glucoside | C21H20O11 | 480-10-4 | 6.08 | 0.001370002 |
| 18 | (+)-Dihydrokaempferol | C15H12O6 | 480-20-6 | 6.44 | 0.116771739 |
| 19 | Resveratrol | C14H12O3 | 501-36-0 | 6.67 | 0.002192 |
| 20 | Daidzein | C15H10O4 | 486-66-8 | 6.83 | 0.000371902 |
| 21 | Luteolin | C15H10O6 | 491-70-3 | 7.03 | 0.007945786 |
| 22 | Quercetin | C15H10O7 | 117-39-5 | 7.07 | 0.085230354 |
| 23 | Hydrocinnamic acid | C9H10O2 | 501-52-0 | 7.13 | 0.454282564 |
| 24 | Trans-Cinnamic acid | C9H8O2 | 140-10-3 | 7.23 | 19.30227412 |
| 25 | Naringenin | C15H12O5 | 480-41-1 | 7.55 | 0.221877642 |
| 26 | Apigenin | C15H10O5 | 520-36-5 | 7.55 | 0.004935392 |
| 27 | Phloretin | C15H14O5 | 60-82-2 | 7.53 | 0.010081457 |
| 28 | Kaempferol | C15H10O6 | 520-18-3 | 7.64 | 0.072625623 |
| 29 | Isorhamnetin | C16H12O7 | 480-19-3 | 7.71 | 0.007630474 |
| 30 | Isoliquiritigenin | C15H12O4 | 961-29-5 | 7.88 | 0.006207853 |

**Table S2.** **Statistical analysis of the number of microbial OTUs at various levels.**

| ID. | domain | phylum | class | order | family | genus | species |
| --- | --- | --- | --- | --- | --- | --- | --- |
| Control1 | 1 | 12 | 17 | 36 | 53 | 104 | 42 |
| Control2 | 1 | 11 | 16 | 31 | 52 | 108 | 38 |
| Control3 | 1 | 13 | 17 | 33 | 49 | 104 | 41 |
| Control4 | 1 | 12 | 17 | 34 | 49 | 108 | 40 |
| Control5 | 1 | 12 | 17 | 34 | 53 | 116 | 44 |
| Control6 | 1 | 14 | 19 | 35 | 53 | 128 | 60 |
| Model1 | 1 | 12 | 16 | 31 | 43 | 96 | 35 |
| Model2 | 1 | 11 | 16 | 32 | 45 | 106 | 35 |
| Model3 | 1 | 10 | 15 | 31 | 45 | 107 | 44 |
| Model4 | 1 | 11 | 16 | 29 | 44 | 106 | 36 |
| Model5 | 1 | 12 | 17 | 32 | 47 | 102 | 37 |
| Model6 | 1 | 12 | 17 | 33 | 47 | 99 | 38 |
| MMF1 | 1 | 15 | 18 | 37 | 54 | 111 | 47 |
| MMF2 | 1 | 13 | 18 | 33 | 44 | 103 | 43 |
| MMF3 | 1 | 12 | 16 | 33 | 49 | 106 | 49 |
| MMF4 | 1 | 12 | 16 | 32 | 51 | 113 | 38 |
| MMF5 | 1 | 13 | 18 | 35 | 55 | 108 | 43 |
| MMF6 | 1 | 17 | 21 | 40 | 60 | 127 | 58 |
